# Clinical Characteristics, Treatment Patterns, and Outcomes of Primary Canaliculitis among Patients in Beijing, China

**DOI:** 10.1155/2015/904756

**Published:** 2015-02-17

**Authors:** Qin Zhang, Beibei Xu, Xiao-Xin Li, Ming-Wu Li

**Affiliations:** ^1^Department of Ophthalmology, People's Eye Institute, Peking University People's Hospital, Beijing 100044, China; ^2^Key Laboratory of Vision Loss and Restoration, Ministry of Education, Beijing 100044, China; ^3^Peking University Center for Human Disease Genomics, Peking University Health Science Center, Beijing 100191, China; ^4^Peking University Medical Informatics Center, Beijing 100191, China

## Abstract

*Background*. Canaliculitis may cause punctal or canalicular swelling, discharge, erythema, and sometimes concretions. This study examined the clinical characteristics, treatment patterns, and outcomes of primary canaliculitis from patients at a top-rated hospital in Beijing, China. *Methods*. Medical records of 16 patients (retrospective case series) were studied. *Results*. This study included four males and twelve females with a median age of 72.5 years. The mean and the median follow-up time were 10.4 months and 6 months, respectively. The mostly observed clinical symptoms were epiphora with discharge (94%), while the mostly observed signs included pouting punctum (75%) and punctal regurgitation of concretions under syringing (75%). Only the symptoms of one patient among those with conservative therapy completely resolved within two-year follow-up. Curettage therapy was found to partly resolve the clinical symptoms and signs within the follow-up of four weeks. Fifteen patients finally received curettage with punctoplasty, and symptoms completely resolved in fourteen patients after one surgery. *Conclusions*. Syringing with pressing of lacrimal sac area may help better diagnosis of canaliculitis. Additionally, curettage with punctoplasty is recommended for thorough removal of concretions and complete resolution of canaliculitis.

## 1. Background

Canaliculitis, as an uncommon infectious eye disease, may cause punctal or canalicular swelling, discharge, erythema, and sometimes concretions [1–3]. This disease is often misdiagnosed for its common symptoms of punctal swelling and no concretions detected by routine clinical examinations with recurrent conjunctivitis, dacryocystitis, or chalazion [1, 2, 4, 5]. So far, no clinical guidelines for canaliculitis have been available to make an accurate diagnosis as well as to provide an effective treatment plan. The previous studies were mostly conducted in one single institute with small sample size, which make the findings difficult to be generalized to a broader population. More studies are needed to establish the clinical guidelines for better diagnosis and treatment of canaliculitis.

The probability of recurrence ranged from 26% to 100% among these canaliculitis patients who presented with concretions [6–8]. In addition, conservative therapy using topical antibiotics was reported to have a high probability of recurrence among canaliculitis patients in previous studies [1, 7, 8]. Canalicular curettage after canaliculotomy or punctoplasty was recommended by previous reports conducted in Australia [1], India [3], and Taipei and China [8] to completely remove canalicular contents and debris [1, 3, 8]. Thorough curettage and curettage after one-snip punctoplasty [6, 9] were recommended to avoid canalicular luminal narrowing or scarring, lacrimal pump dysfunction, or canalicular fistula formation after canaliculotomy [7, 10]. However, other studies suggested that canaliculotomy may help curettage of the canalicular contents and would not cause the epiphora after canaliculotomy [1, 7, 11, 12]. To our knowledge, few studies compared curettage therapy only and curettage with punctoplasty. Herein, this study examined the clinical characteristics and treatment patterns and compared different treatment plans of primary canaliculitis among Chinese patients at a top-rated hospital in Beijing, China.

## 2. Methods

This study included sixteen patients diagnosed with primary canaliculitis between April 1, 2010, and September 30, 2012, at Peking University People's Hospital, one top-rated hospital for lacrimal diseases. The review protocol for this retrospective study was approved by the Institutional Review Board of Peking University People's Hospital.

The canaliculitis is diagnosed by mucopurulent punctal regurgitation or concretions extruding from the punctum associated with eyelid thickening or eyelid erythema, which were based upon previous reports [1–3, 8, 13]. Canaliculitis that arose from punctual plug placement or nasolacrimal duct obstruction was considered as a secondary canaliculitis and thus was not included in this study. Data were obtained from patients' medical records including demographic characteristics, symptoms and signs, comorbidities, time between onset of symptoms and diagnosis, side and location of involvement, presence of concretions, treatment, and outcomes.

**Figure 1:**
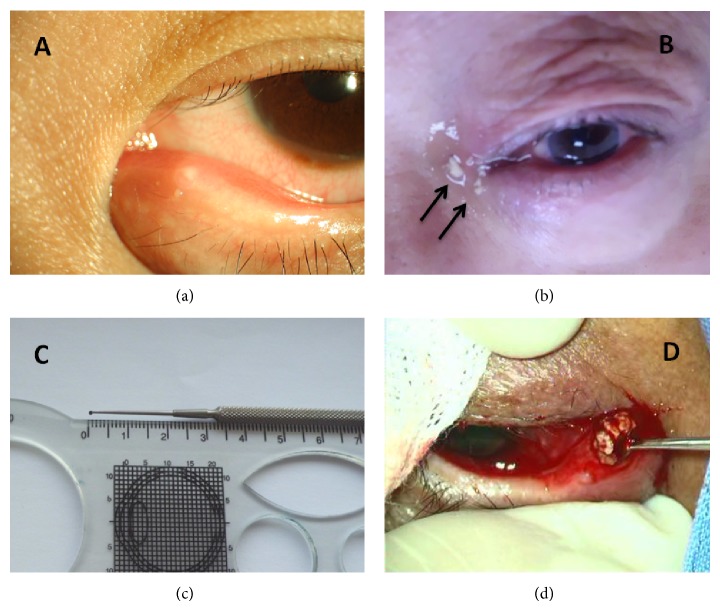
The clinical signs and procedures of canaliculitis. (a) Classic pouting punctum; (b) punctal regurgitation of concretions under syringing; (c) the 0.9 mm curette for single curettage; (d) removal of concretions in surgery. Arrow: the sulphur granules.

The treatment plans included conservative treatment (canalicular expression by a tweezer, topical povidone-iodine syringing, and topical antibiotics) and surgery (single canalicular curettage or 2-snip punctoplasty and curettage). Outcomes included complete resolution, recurrence of symptoms and/or signs, and partial resolution. Complete resolution was defined as the disappearance of all clinical symptoms and signs after the treatment during the follow-up [3]. Recurrence was defined as the reemergence of clinical symptoms and signs during the period of follow-up [3]. Partial resolution was defined as remaining clinical symptoms and signs. Conservative treatment was performed under 1% tetracaine hydrochloride topical anesthesia. A wild tweezer was used for complete expression of purulent discharge and concretions, which were sent for further microbiological culture and histopathological examination. Povidone-iodine was used with syringing before and after expression or without expression of canalicular contents. Patients were prescribed antibiotic eye drops (Levofloxacin or Tobramycin) 4 times per day after each follow-up examination. To avoid antibiotic resistance, each type of antibiotic eye drop was used for no more than one month. Canalicular curettage was performed under local infiltrative anesthesia with 1% lidocaine hydrochloride injection. Curettage was performed with a 0.9 mm curette (Figure 1(c)). After dilatation of the punctum with a punctum dilator, the curette was used to completely remove the granulation tissue and concretions. This procedure was repeated once a week whenever concretions or discharge was observed during the follow-up visit in one month. Punctoplasty and canalicular curettage were performed under the same anaesthetic procedure. A 2-snip punctoplasty was performed with straight Vannas scissors, and a 2 mm chalazion curette was used to completely remove the granulation tissue and concretions, which were sent for further microbiological culture and histopathological examination.

## 3. Results

Descriptive characteristics of the patients were presented in Table 1. Twenty-one outpatients were diagnosed with primary canaliculitis. After excluding five patients with unavailable follow-up information, sixteen patients were finally included (Table 1). There were four (25%) male and twelve (75%) female participants with a median age of 72.5 years (range: 50–85 years) and a median follow-up time of 6 months (range: 3–34 months). The median time from the onset of symptom to diagnosis was 18 months (range: 0.25–48 months). All patients were presented with unilateral eye involvement. The left eye was involved in ten patients (62.5%) while the right was involved in six patients (37.5%). The upper canaliculus was affected in eight patients (50%), the lower canaliculus was affected in six patients (37.5%), and both upper and lower canaliculi were affected in two patients (12.5%). The primary symptom was epiphora with discharge (93.8%), followed by redness (31.3%), swelling (12.5%), and pain (6.3%). The primary clinical sign was pouting punctum (75%) (Figure 1(a)); other signs were a palpable thickened canaliculus (50%) and punctal regurgitation of canalicular contents under expression (31.3%). However, canalicular contents were easier to see while syringing (75%) (Figure 1(b)). Lacrimal was patent after syringing in 15 patients (93.8%).

Microbiologic examinations were performed on samples of canalicular contents from six patients.* Staphylococcus* species were isolated from two of these patients. These two patients both firstly received conservative treatments, including antibiotics and expression for 4 weeks, and did not completely resolve. One patient subsequently received curettage with punctoplasty and resolved completely. The second patient received a second course of single curettage for four weeks but still failed to resolve and therefore underwent curettage with punctoplasty, after which this patient resolved completely. Those four patients with negative microbiologic results all firstly underwent conservative therapy and only one of them completely resolved. For the remaining three patients who did not resolve completely, two of them finally resolved completely using curettage with punctoplasty, and the last one received a second course of single curettage for four weeks while this patient resolved completely using curettage with punctoplasty.

Six patients received dacryocystography. Four patients had dilatation and/or roughness of the wall of canaliculus, and of these three patients showed filling defects at the angle of the canaliculus irrespective of whether the two upper canaliculi or one lower canaliculus was involved (Figure 3(a)). Two patients retained the residue of the contrast agent for 20 minutes after injection (Figure 3(b)). The photomicrograph with Gomori methenamine silver stain showed the presence of* Actinomyces*-like filaments in the concretions from one patient.

**Figure 2:**
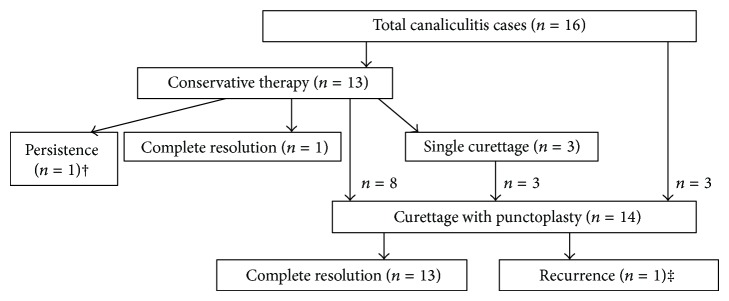
Flow diagram of sample size for treatments and outcomes. †: this patient declined subsequent therapy after conservative therapy but kept long-term follow-up appointments. The condition was found to be persistent in this patient. ‡: this patient received conservative therapy first and then received curettage with punctoplasty directly.

The treatment selection was summarized in Figure 2. Thirteen patients received conservative therapy at the time of the first visit. Only one of them resolved completely. Another one declined subsequent surgery (curettage or punctoplasty and curettage) and did not resolve thereafter. The remaining eleven patients were treated with conservative therapy and had no improvement or only partial remission of the condition. Three of these patients received curettage with a 0.9 mm curette. Partial remission of symptoms and signs were found at each weekly visit in the four-week follow-up, and thus patients received curettage therapy at each of the four visits. However, complete resolution of canaliculitis was not observed in any of these patients. Since curettage only cannot resolve this disease, the three patients received curettage and punctoplasty after one-month curettage therapy. Eight of eleven patients failed in conservative therapy and received punctoplasty and curettage thereafter (Figure 1(d)). Seven of these patients experienced complete resolution of symptoms and signs of canaliculitis with one surgery. Recurrence of symptoms and signs was observed in one of the eight patients two months after surgery. Punctoplasty and curettage were not performed again at the patient's request. Three patients received punctoplasty and curettage after diagnosis of canaliculitis directly; all of these patients experienced complete resolution of symptoms and signs of canaliculitis.

## 4. Discussion

Our study reported that the median time to diagnosis of canaliculitis was 18 months and the numbers in other studies varied from 4.5 months to 34 months [2, 3, 6, 8, 9, 14, 15]. It is difficult to explain the large variation for different studies because all studies were conducted in different locations with small sample size. The mean age of patients was 70.6 years in our study while other studies reported the mean age to be ranging from 48 to 71.7 years [1–3, 6–9, 14, 15]. Similar to finding from other studies, most canaliculitis patients in our study were females (75%) [1, 3, 8, 9]. This may be explained by hormonal changes during menopause which will decrease the tear production and reduce the protection against infections [1]. Single upper canaliculus was affected in half of the patients while single lower canaliculus was affected in less than half of the patients, which differs from most published data [3, 6–9, 14–16]. Similar to other studies, we also observed epiphora with discharge as most common symptom (93.8%) and pouting punctum as the most common sign (75%) [2, 3, 6–9, 14]. Our study found that 31% and 75% of the study patients had concretions from the procedure of expression and syringing, respectively. Pavilack and Frueh reported that all eleven study patients had concretions in the expressed mucopurulent material [6]. Using a different procedure of curettage other than expression or syringing, Anand reported that 33% of the 15 study patients had concretions [7]. In Lin's study, concretions were obtained during canalicular compression or canaliculotomy in 9 of 34 patients (26%) [8]. Some reports mentioned that the presence of concretions in canaliculus indicated a risk of failure for conservative therapy and a risk for recurrence of canaliculitis [4, 6–8, 14, 17–19]. It is important to examine the presence of concretions in canaliculus which will help make decisions for treatment plans. We found syringing can help to better detect the presence of concretions. When lacrimal syringing was patent in nearly all patients (93.8%), syringing with pressing of lacrimal sac area is helpful for the regurgitation and detectionof canalicular contents. Concretions were found in 12 patients with syringing with pressing of lacrimal sac area in this study. Therefore, syringing not only is a method of treatment but also will help make an accurate diagnosis for canaliculitis. Similar to Lin's study, we also found the patency of lacrimal passage [8].* Actinomyces* was suggested to be the pathogenic bacteria of canaliculitis [6, 11, 12, 19, 20]. Results of this study show that samples from six patients were examined microbiologically and of these, samples from only two patients were positive for the presence of* Staphylococcus* species. The pus and canalicular contents collected by syringing were used for culture, and the low rate of positive bacterial cultures (33%) may be due to the specimen selection. In some reports, routine conjunctival eye swabs were not ideal for microbiological culture [19] while other reports indicated that the low incidence of the isolation of* Actinomyces* may be due to its fastidious nature [7]. The photomicrograph with Gomori methenamine silver stained showed* Actinomyces*-like filaments that might be evidence of the presence of* Actinomyces* in concretions (Figure 4).

**Figure 3:**
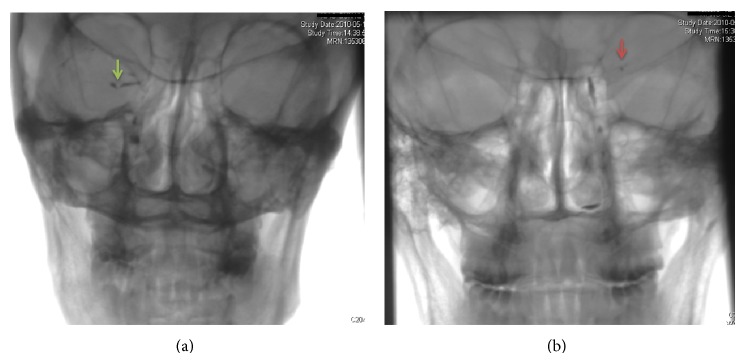
The filling defect of canaliculus with dilation (a) and the residue of the contrast medium (b) presented in the dacryocystography of canaliculus patients. Green arrow: the filling defect of the canaliculus. Red arrow: the residue of the contrast agent.

**Figure 4:**
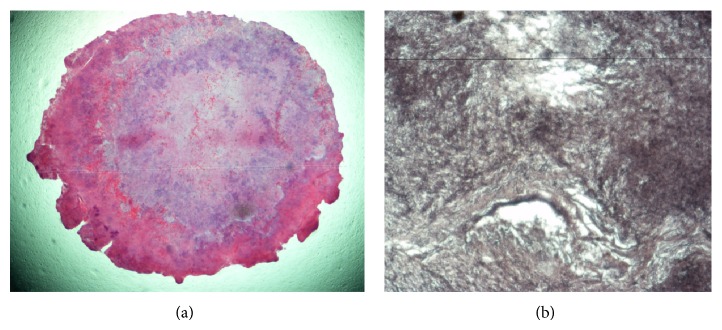
The photomicrograph of concretion. (a) The photomicrograph of a cross section of concretions (stain, hematoxylin-eosin; original magnification, ∗40x). (b) The photomicrograph showing* Actinomyces*-like filaments within the concretion (stain, Gomori methenamine silver; original magnification, ∗400x).

Dacryocystography may not be necessary for diagnosis, while it is helpful for locating the lesion. Of six patients receiving dacryocystography, dilatation and/or roughness of the wall of canaliculus were observed among four patients and filling defects (Figure 3(a)) for three patients at the angle of the canaliculus. The residue of the contrast agent in canaliculus was found in two patients for 20 minutes after injection. Demant and Hurwitz also suggested that dilatation and raggedness of the canaliculus are associated with canaliculitis [12]. Filling defects of the canaliculus are considered to be diagnostic of canaliculitis by Sathananthan et al. [21]. Our results here showed that all filling defects were located near the angle of canaliculus. The angle of canaliculus may tend to be the site of the original lesion, which is different than the results of a previous study which reported that “although a diverticulum or obstruction of the canaliculus can promote anaerobic bacterial growth secondary to stasis, most cases of canaliculitis originate without any identifiable predisposition” [3]. Considering the patency of lacrimal passage and the location of the lesion, hydrodynamic factors may lead to the deposition of pathogenic microorganisms near the angle of the canaliculus.

Conservative therapy was ineffective in this study except for one patient with no concretions observed from both expression and syringing. The low rate of complete resolution of canaliculitis using conservative therapy may be related to the presence of concretions. Pavilack and Frueh suggested that thorough curettage without punctoplasty could be adequate for complete curettage [6]. We tried the curettage, a low-cost approach, for three of our patients after conservative treatment failed. All three patients experienced partial remission of symptoms and signs in one or two weeks, but their conditions did not completely resolve. We used a 0.9 mm closed curette rather than the 1 to 2 mm closed curette used in Pavilack's study [6]. The curette with a small size may be insufficient to completely remove the canalicular contents while the larger size requires a perfect dilatation of punctum, which is difficult to accomplish in surgery. It is difficult to remove all the canalicular contents using curettage, especially for the inexperienced surgeon [11]. Canaliculitis was recurrent in only one of 14 patients in two months using curettage with punctoplasty. This high rate of complete resolution may be due to the complete removal of concretions during surgery [6, 7, 19].

Different from previous studies, we did not observe the gender difference for the recurrence of canaliculitis and dacryoliths. Our study found that the key factor to influence the treatment outcomes is the detection of concretions. There is a strong correlation between the presence of concretions and the failure of conservative therapy. Surgery, as a better procedure to completely remove the canalicular contents, may be preferred to treat canaliculitis patients with concretions.

There were some limitations for this study. First, the sample size was small which could limit the generalization of the results. However, we noticed that canaliculitis is a rare disease and all previous studies were single institution analyses with very small sample size. In addition, the microbiologic examination was performed in six patients, and samples with concretions found during surgery would be more likely to harbor microorganisms that might be cultured. Fourth, limited demographic information was available to understand the potential risk factors of this rare disease. Future studies with a larger sample size, a longer follow-up, and more information collected may be helpful to understand this uncommon disease.

## 5. Conclusion

In summary, oculists should avoid misdiagnosis of canaliculitis to prevent delays in treatment. Our study suggested that syringing with pressing of lacrimal sac area may help better diagnosis of canaliculitis. Because the majority of canaliculitis patients had patent lacrimal passage, water may flow into lacrimal sac and then into the nasal cavity by routine syringing. Pressing of lacrimal sac area keeps the water fully washing canaliculus and thus gets better regurgitation and detection of canalicular contents. With the symptoms/signs of epiphora, discharge, and punctal swelling observed, canaliculitis should still be suspected even with no concretions observed among patients with patent lacrimal duct. Additionally, compared to syringing, expression, and curettage, curettage with punctoplasty is more reliable for thorough removal of concretions and complete resolution of canaliculitis.

## Figures and Tables

**Table 1:** Clinical characteristics, treatments, and outcomes for 16 patients diagnosed with canaliculitis.

	Median (25th percentile, 75th percentile)
Age (years)	72.5 (63.8, 77.5)
Follow-up (months)	6 (5, 12)
Time to diagnosis (months)	18 (10, 24)

	*N* (%)

Gender	
Male	4 (25)
Female	12 (75)
Coexisting diseases	
Diabetes mellitus	4 (25)
Hypertension	8 (50)
Symptoms	
Epiphora with discharge	15 (94)
Redness	5 (31)
Swelling of the eyelid	2 (13)
Pain	1 (6)
Clinical signs	
Pouting punctum	12 (75)
Palpable thickened canaliculus	8 (50)
Punctal regurgitation of concretions under expression	5 (31)
Punctal regurgitation of concretions under syringing	12 (75)
Location	
Upper canaliculus only	8 (50)
Lower canaliculus only	6 (38)
Both	2 (13)
Laterality	
Right	6 (38)
Left	10 (63)
Presence of concretions	15 (94)
